# Military Medical Officers

**Published:** 1916-03

**Authors:** 


### BUFFALO MEDICAL JOURNAL

**A Monthly Review of Medicine and Surgery**

EDITOR AND PUBLISHER, DR. A. L. BENEDICT, 228 Summer St., corner of Elmwood Ave., Buffalo, (Address for all communications. Please make personal and telephone calls before 1 P. M.)

Third, in age, on the western continent. Independent, but supports professional organization. News limited mainly to Western N. Y. and Penn.

Subscription $2.00 a year; $3.00 for two years in advance. Changes and errors in address or failure or duplication of delivery, should be reported immediately.

Yearly Volume 71 MARCH, 1916 No. 8

### Military Medical Officers

Aside from the provision of materials of war, now complicated and costly, the great problem of military preparedness is correlation. It does not matter much whether the manual of arms and evolutions are perfectly performed or even whether the individual soldier shoots accurately or not—except in the case of artillery for which regular training and more particularly, applied science are necessary. No one detail of preparedness so far as correlation is concerned, is of great difficulty but each is tedious and all must be worked out with reference to mutual harmony. A considerable period of time is necessary to convert the abundance of patriotic and intelligent men who constitute our militia in the proper sense, into an adequate defensive force.

We call attention to one detail in which the medical profession is especially interested. A vast amount of the work of the medical staff is medical in the restricted sense, involving physical examination, advice as to hygiene and sanitation not in any way approaching the technical details of physical training for strictly military purposes nor the formulation of rules for camp and trench life, and there still remains a very large amount of purely medical practice. A great many physicians are willing and anxious to perform such services in case of need and to prepare for an emergency by national guard or auxiliary service in peace but they are neither surgeons, bacteriologists nor sanitary experts. They have no time to prepare for an examination which will enroll them as surgeon in theory nor do they wish, even nominally, to pose as surgeons, bacteriologists or sanitary experts, much less to do actual damage by attempting to discharge such duties under orders based on the assumption that they are anything else than medical practitioners. This fear may be imaginary. Our predecessor, the late Lt. Col. W. W. Potter, M. D., in describing the surgical service of the Civil War, emphasized the fact that details for operative work were based on actual proficiency, without regard to rank. But, from the opposite standpoint, it may not be a groundless fear that personal ambition may lead to the seeking of surgical experience by men not properly qualified.

Be this as it may, the fact remains that, in peace, the work of the army surgeon is mainly non-surgical, and that in war, there will be, on the one hand, a large demand for distinctly surgical work and, on the other, for an approximately equal amount of distinctly medical work. Under present conditions, it is rarely that the surgeon is a good internist or the internist a good operator. This is partly due to the fact that the two branches of the medical art call forth quite different forms of ability, even more due to the fact that very few men have the time and strength to perfect themselves in both.

The time is ripe to recognize that these facts present definite conditions which must be met to insure preparedness in the military sense, even with regard solely for military efficiency and not for humanity or the after-effects of war. In Europe, practically all practitioners are ipso facto, members of the medical corps of the army, with training in time of peace and billeted provisionally, for any demand that may come upon their country, according to their individual abilities. In this country, it is probable that voluntary service in all branches of the military organization, will suffice but there should be devised a practical plan for providing both surgeons and physicians and such scientific and practical specialists as are required, with proper allotment of duties and with equal rank, according to duration and value of services, just as in the different arms of the fighting service. And, the time to provide for this adjustment to modern conditions and possible future needs, is now.

